# Chlamydial Entry Involves TARP Binding of Guanine Nucleotide Exchange Factors

**DOI:** 10.1371/journal.ppat.1000014

**Published:** 2008-03-07

**Authors:** B. Josh Lane, Charla Mutchler, Souhaila Al Khodor, Scott S. Grieshaber, Rey A. Carabeo

**Affiliations:** 1 Department of Microbiology and Immunology, University of Louisville Medical School, Louisville, Kentucky, United States of America; 2 Department of Oral Biology, University of Florida School of Dentistry, Gainesville, Florida, United States of America; Duke University Medical Center, United States of America

## Abstract

*Chlamydia trachomatis* attachment to cells induces the secretion of the elementary body–associated protein TARP (Translocated Actin Recruiting Protein). TARP crosses the plasma membrane where it is immediately phosphorylated at tyrosine residues by unknown host kinases. The Rac GTPase is also activated, resulting in WAVE2 and Arp2/3-dependent recruitment of actin to the sites of chlamydia attachment. We show that TARP participates directly in chlamydial invasion activating the Rac-dependent signaling cascade to recruit actin. TARP functions by binding two distinct Rac guanine nucleotide exchange factors (GEFs), Sos1 and Vav2, in a phosphotyrosine-dependent manner. The tyrosine phosphorylation profile of the sequence YEPISTENIYESI within TARP, as well as the transient activation of the phosphatidylinositol 3-kinase (PI_3_-K), appears to determine which GEF is utilized to activate Rac. The first and second tyrosine residues, when phosphorylated, are utilized by the Sos1/Abi1/Eps8 and Vav2, respectively, with the latter requiring the lipid phosphatidylinositol 3,4,5-triphosphate. Depletion of these critical signaling molecules by siRNA resulted in inhibition of chlamydial invasion to varying degrees, owing to a possible functional redundancy of the two pathways. Collectively, these data implicate TARP in signaling to the actin cytoskeleton remodeling machinery, demonstrating a mechanism by which *C. trachomatis* invades non-phagocytic cells.

## Author Summary

The human pathogen *Chlamydia trachomatis* is the causative agent of the most prevalent bacterial sexually transmitted disease in industrialized nations, and of the preventable blinding condition trachoma in developing countries. Survival and replication of chlamydial species occur exclusively inside a host cell, and thus, gaining access to the protective intracellular niche is an absolute requirement. This report describes how the chlamydia protein TARP, which is secreted at the base of the bacteria and across the host membrane, acts as a scaffold to which host signaling proteins bind. This assembly of the complex of signaling proteins, which include Sos1, Abi1, Eps8, and Vav2 results in the remodeling of the host cytoskeleton to facilitate engulfment of the infecting chlamydia. We conclude that these proteins have a role in chlamydia based on a number of observations including their interaction with the TARP protein, their ability to switch on known signaling participants in chlamydia invasion, their localization at the site of chlamydia entry, and the inhibition of chlamydia invasion in their absence. Altogether, the data functionally link TARP with signaling pathways that function in chlamydial invasion, demonstrating the direct involvement of TARP in the invasion of host cells by *C. trachomatis*.

## Introduction

Chlamydiae are obligate intracellular bacterial pathogens that are responsible for a number of human diseases [1]. Different serological variants of *Chlamydia trachomatis* are primarily pathogens of humans. Serological variants (serovar) A, B, Ba, and C are the etiologic agents of trachoma, the leading cause of preventable blindness worldwide. Serovars D to K are associated with sexually transmitted diseases, and serovars L1, L2, and L3 cause lymphogranuloma venereum, a more invasive sexually transmitted disease. *Chlamydophila psittaci* is a zoonotic agent that causes a pneumonia-like respiratory disease in humans. *Chlamydophila pneumoniae* is a causative agent of community-acquired pneumonia, and has recently been associated with cardiovascular diseases. The genii Chlamydia and Chlamydophila share several biological properties, including a biphasic developmental cycle that includes cell types adapted for extracellular survival (elementary bodies or EBs) or intracellular multiplication (reticulate bodies or RBs) [2]. Intracellular development occurs within a protective vacuole called an inclusion, which is nonfusogenic with endocytic vesicles, but is instead interactive with an exocytic pathway that delivers sphingomyelin and cholesterol from the Golgi apparatus to the inclusion [3].

Because of the obligate intracellular nature of chlamydiae, access to the inside of the cell is paramount to survival. To this end, Chlamydiae have evolved to efficiently invade non-phagocytic cells through a process that has been described as “parasite-specified phagocytosis” [4]. Because entry into host cells is a critical step in the chlamydial developmental cycle, this stage of infection is an especially attractive chemotherapeutic target for inhibition. Thus, considerable efforts have been put forth to understand the molecular mechanism of chlamydial invasion. Several chlamydial ligands and host receptors have been proposed, although there has been little consensus as to which of the number of chlamydial ligands and host receptors are of primary importance. It is likely that many of these ligand-receptor interactions function in infection of different cell and tissue types conferring an advantage during infection [5].

Chlamydial invasion is initiated by the electrostatic and reversible interaction of EBs mediated through host heparan sulfate-like proteoglycans, followed by an irreversible host-dependent step that leads to internalization of EBs [6]–[8]. While the identity of the host factors in this secondary irreversible step has yet to be identified, the characterization of the molecular mechanism of the post-attachment stages of chlamydial infection is beginning to be defined [7], [9]–[12]. Upon attachment of EBs, there is a demonstrable rapid recruitment of actin at the sites of attachment, leading to the the formation of pedestal-like structures underneath attached EBs [9],[11]. This recruitment of actin is transient and eventually leads to the uptake of EBs into membrane-bound vesicles that are devoid of known early endosomal markers [13].

Recently, a chlamydial protein associated with the uptake of EBs was found to be translocated by a type III secretion system into the host cell at the site of entry [14]. Once in the cytosol, the protein quickly becomes tyrosine phosphorylated by host kinases. This protein, termed TARP for translocated actin-recruiting protein, is likely to be involved in chlamydial invasion in that it is able to interact with both filamentous and monomeric actin [15]. Interestingly, live cell imaging studies demonstrated tyrosine phosphorylation preceding actin recruitment, leading to the hypothesis that TARP plays a key role in initiating a signal transduction cascade that leads to the activation of the cellular actin remodeling machinery [14]. A striking feature of the TARP protein is the presence of several tyrosine-rich tandem repeats of approximately 50 amino acids in length. The number of repeats differs, with *C. trachomatis* urogenital isolate, serovar D, containing three repeat units and an LGV strain with almost six full repeat units [16]. Some isolates lack these repeats, and are also able to recruit and remodel actin to facilitate their invasion [11]. This is consistent with recent functional studies of TARP that concluded a C-terminal domain located downstream of the tandem repeat region contributes to actin recruitment and nucleation [15]. Here we report of an alternate mechanism of actin remodeling by TARP that involves the repeat domain. Our data show that phosphorylation of critical residues in this region initiates a signal transduction cascade by interacting with guanine nucleotide exchange factors, Sos1 and Vav2. Mutations in the relevant tyrosine residues resulted in the loss of the ability of TARP to interact with these proteins, preventing recruitment of Rac and actin, and reduced invasion.

## Results

### A single phosphodomain unit of TARP functions to recruit actin and Rac

**Figure 1:**
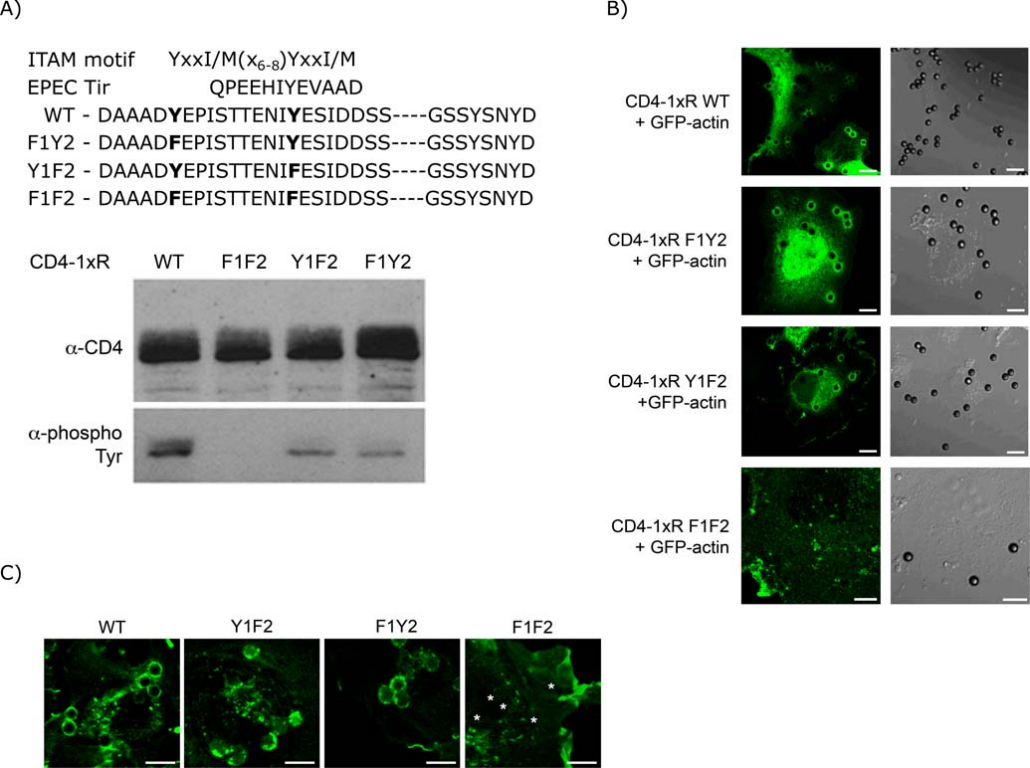
Recruitment of GFP-actin after binding of 40 µm beads coated with α-CD4 antibody to cells expressing the fusion protein CD4-1xR and its mutant derivatives. a) The wild-type and mutant sequence of the second repeat of the TARP protein from the *C. trachomatis* serovar L2 is shown, from amino acid 174 to 222, the amino acid sequence of the Immunoreceptor Tyrosine-based Activator Motif (ITAM), and the surrounding amino acids of the tyrosine residue in the Tir protein essential for actin pedestal formation by EPEC to highlight the potential for this region of the TARP protein to be recognized by signaling molecules that are known to participate in actin cytoskeletal remodeling. Relevant tyrosine and substituted phenylalanine residues are in bold. Western blot of total lysates prepared from cells transfected with the various CD4-1xR expression constructs demonstrates equal levels of expression of the different derivatives of the CD4-1xR fusion protein and their respective reactivity to the 4G10 anti-phosphotyrosine antibody; b) Recruitment of GFP-actin upon aggregation of the plasma membrane-localized CD4-1xR fusion derivatives after incubation with beads coated with the monoclonal anti-CD4 antibody. Note the lack of GFP-actin recruitment in the F1F2 mutant, indicating the requirement for the presence of at least one of the two tyrosine residues within the ITAM sequence. c) Localization of GFP-Rac at the sites of CD4-1xR aggregation by the anti-CD4 antibody-coated beads. Note the lack of GFP-Rac recruitment in the F1F2 mutant, indicating the requirement for the presence of at least one of the two tyrosine residues within the ITAM sequence. In addition, membrane ruffles could be observed, which is typically found in cells that overexpress Rac. Asterisks indicate the position of the beads. Scale bars = 10 µm

Upon interaction of the EBs with epithelial cells, the TARP tyrosine residues that are phosphorylated are within the context of the phosphorylation sites for members of the Src-family of kinases and recognition sites of various src-homology 2 domain (SH2)-containing adaptor proteins (Figure 1a). The presence of these sequences raises the possibility that TARP may recruit signaling molecules that recruit and remodel actin. To directly test this hypothesis, a mammalian expression vector with an insert that encodes for a fusion protein containing the N-terminal extracellular domain of CD4 (amino acids 1–372) and one phosphodomain unit, with wild type or mutant sequences was synthesized, and co-transfected into Cos-7 cells along with either GFP-actin or GFP-Rac1. The second repeated (amino acids 174–222) unit has the sequence DAAADYEPISTTENIYESIDDSSTSDPENTSGGAAALNSLRGSSYSNYD, with the relevant tyrosines underlined (Figure 1a). These tyrosine residues were targeted because they are in the context of the recognition motifs for various Src kinase family and SH2 domain-containing adapter proteins [17]. Interestingly, these features of the tyrosines in the TARP phosphodomain are shared by the critical tyrosine in the Tir protein of enteropathogenic E. coli [18].

Expression of the fusion proteins was allowed to continue for 24 h post-transfection. The cells were incubated for 2 h with 4.5 uM beads coated with anti-CD4 antibody molecules to induce aggregation of plasma membrane-localized CD4-1xR fusion proteins. The highly localized tyrosine phosphorylation [14] and subsequent formation of actin-rich structures (i.e. pedestal and microvilli) directly underneath the attaching EB particle [9] are indicative of a signaling process that is restricted to the area directly underneath the invading EB particle. Indeed, the re-initiation of the formation of these actin-rich structures after disruption by the actin-destabilizing agent cytochalasin D was preferentially localized to the sites of chlamydia attachment (Figure S1). The induced aggregation provided defined spaces in which to monitor the recruitment of GFP-actin and/or GFP-Rac1. Doubly transfected cells were decorated with the beads and appeared green under fluorescence microscopy. For those transfected with CD4-1xR (WT), a ring-like localization of GFP-actin could easily be seen surrounding the beads. CD4-1xR (Y1F2) and (F1Y2), in which the tyrosines were mutated to phenylalanines, also demonstrated recruitment of GFP-actin and GFP-Rac1, while the double mutant CD4-1xR (F1F2) failed to show the same recruitment (Figure 1a). The differences in the ability to recruit actin and Rac1 was not due to differences in expression level as all constructs were expressed equally well (Figure 1b).

The tyrosine residues in the CD4-1xR fusion protein that were actually phosphorylated *in vivo* were determined to be Tyr179 or Tyr189, using a numbering system that starts with the Asp174 residue of the second repeat of the serovar L2 TARP homolog. Figure 1b shows that the mutation of the two tyrosines to phenylalanines (F1F2) eliminated any reactivity with the 4G10 anti-phosphotyrosine antibody. Thus Tyr179 and Tyr189, but not Tyr218 and Tyr221 were phosphorylated. The doublet observed in the lane marked WT was likely due to the singly and doubly phosphorylated forms. Thus, one unit of the phosphodomain of TARP is functional, and that recruitment of actin and Rac1 appeared to require at least one tyrosine to be phosphorylated.

### The oligopeptide spanning a single copy of the repeated units of TARP binds Rac-specific GEFs in a phosphorylation-dependent manner

**Figure 2:**
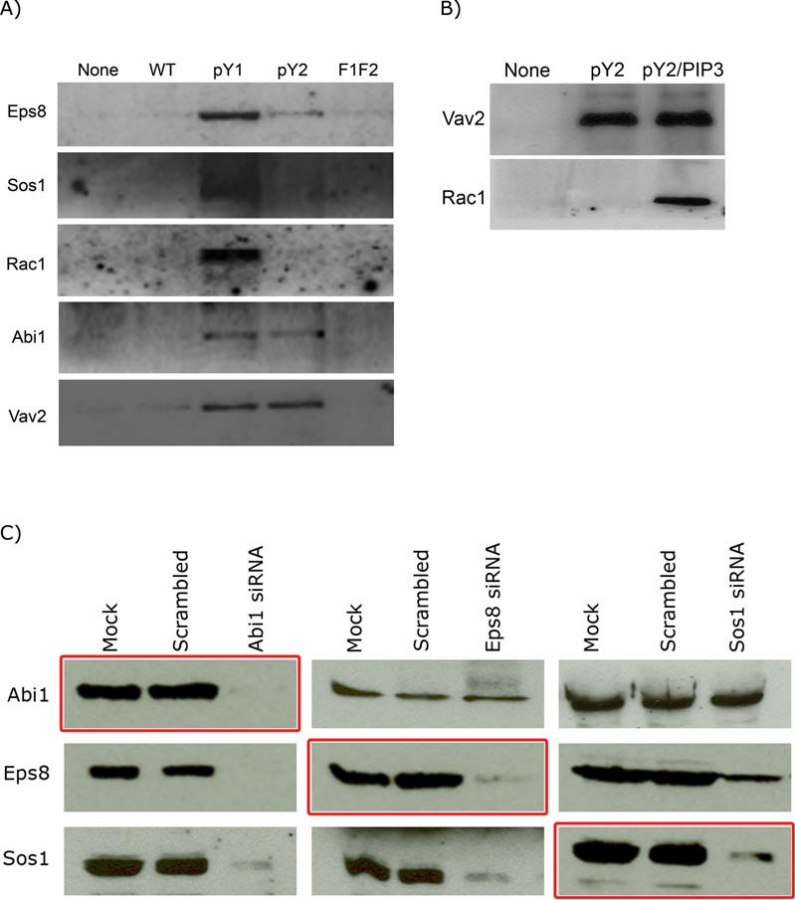
Characterization of the fraction precipitated by the various biotinylated oligopeptide derivatives. a) Western blot of the precipitated fractions to demonstrate the presence of various host proteins that participate in guanine nucleotide exchange of the Rac GTPase. HeLa cell lysates were incubated with the different phosphorylation derivatives of an oligopeptide that spans one unit of the repeated domain. Note the differences in the proteins pulled down by the different oligopeptides. b) The addition of the PI 3,4,5-P_3_ analog to the lysate prior to pulldown enhanced the interaction of Vav2 with Rac1. The intramolecular interaction of Vav2 blocks the binding domain of Rac1, which the lipid analog unmasked, and thus allowed for binding of Rac with Vav2. c) The interaction of the Sos1/Abi1/Eps8 complex with TARP requires the Abi1 protein. Lysates depleted of the various components of this complex were subjected to co-precipitation with the pY1 oligopeptide to determine the nature of its interaction with TARP. Abi1 was required for the coprecipitation of Sos1 and Eps8 with the pY1 oligopeptide, while Sos1 or Eps8 depletion did not affect the interaction of Abi1 with pY1, but rather partially inhibited their respective interactions with pY1. The red borders indicate the blots demonstrating efficiency of siRNA depletion, while the lack of the border signified the coprecipitated levels of the proteins.

Because one copy of the repeated unit is apparently sufficient to recruit actin and Rac1 in our cell culture assay, and the dependence of these activities on the phosphorylation of Tyr179 and Tyr189, it is hypothesized that the domain may act as a signaling platform to which host signaling molecules are recruited. To directly test this possibility, biotinylated oligopeptides with the sequence DAAADYEPISTTENIYESIDDSSTSDPENTSGGAAALNSLRGSSYSNYD were custom synthesized either as non-phosphorylated tyrosines (WT), individually phosphorylated tyrosines (pY1 and pY2), or in which phenylalanines have been substituted for the tyrosines (F1F2). The biotinylated oligopeptides were incubated with lysates from HeLa cells and the presence of molecules known to participate with Rac1 in signal transduction pathways, specifically the Rac guanine nucleotide exchange factors Vav2 and Sos1 were monitored by Western blotting. The specificity of Sos1 towards Rac1 is conferred by the Abi1 and Eps8 proteins [19]. Sos1 exclusively bound to the phosphorylated pY1 peptide, as shown in Figure 2a, while Abi1 bound equally well to pY1 and pY2 oligopeptides. Vav2 bound equally well to pY1 and pY2. Pulldown samples from the WT and F1F2 oligopeptides showed background or undetectable levels of the all the proteins monitored, indicative of the requirement for phosphorylated tyrosine residues.

Interestingly, Rac was only detected in the pY1 fraction, coinciding with the presence of Sos1, Abi1, and Eps8 in the same fraction. While Vav2 was also present in the pY1, it is unlikely that Rac is binding to this protein, because Rac was not pulled down by pY2 despite the presence of Vav2. However, it has been reported previously that Rac association with Vav2 could be enhanced by the addition of PI 3,4,5-P_3_ [20]–[22]. Therefore, a water-soluble analog of this phospholipid was added to the lysates, and the pulldown fraction was assayed for the presence of Rac (Figure 2b). When lysates were pre-treated with the PI 3,4,5-P_3_ analog, the Rac GTPase protein could be detected from the pulldown fractions from both pY1 and pY2. Thus, it appears that interaction of Rac with the phosphodomain of TARP via Vav2 required PI 3,4,5-P_3_.

A series of lysates was prepared from HeLa cells depleted of Sos1, Abi1, and Eps8, and the co-precipitation of these signaling molecules was performed to determine which proteins are required for the interaction of the complex with the pY1 oligopeptide (Figure 2c). The depletion of the Abi1 protein markedly affected the levels of Eps8 and Sos1 coprecipitated by the pY1 oligopeptide, while neither the depletion of Eps8 nor Sos1 significantly affected the level of coprecipitated Abi1 protein. However, depletion of Eps8 negatively affected the ability of Sos1 to be coprecipitated. Sos1 depletion also had a negative effect on Eps8 pulldown. Taken together, the depletion and pulldown data indicate that Abi1 binding to TARP, which is likely to be indirect due to the lack of any SH2 domain, mediated the coprecipitation of Eps8 and Sos1 in a complex. Interestingly, Sos1 and Eps8 may stabilize each other's association with the complex as depletion of Sos1 decreased the coprecipitated levels of the Eps8 and *vice versa*.

### Localized synthesis of phosphatidylinositol 3,4,5-triphosphate during chlamydial invasion

**Figure 3:**
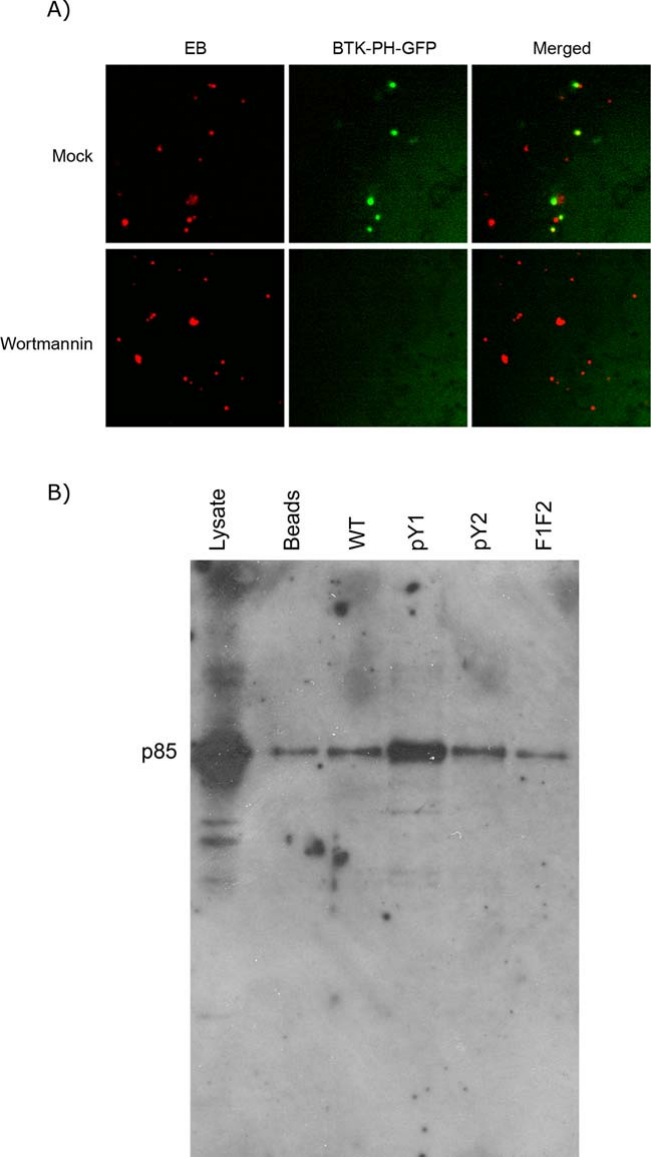
The p85 subunit of phosphatidylinositol 3-kinase interacts with the phosphodomain of TARP. a) Binding of CMTPX-labeled *C. trachomatis* L2 EBs to cells expressing the PI 3,4,5-P_3_ probe GFP-BTK-PH induces the localized synthesis of the lipid, and thus recruitment of the fluorescent probe. The images were obtained by live cell microscopy at 5 sec intervals. Treatment with the PI_3_-kinase inhibitor, wortmannin (100 nM) abolished PI 3,4,5-P_3_ synthesis, demonstrating the specificity of the GFP-BTK-PH probe. B) Oligopeptide pulldown using HeLa cell lysates demonstrate the interaction of the SH2 domain-containing p85 subunit of PI3-kinase in a phosphorylation-dependent manner, showing preference for the pY1 oligopeptide.

Because the presence of PI 3,4,5-P_3_ appears to be necessary for optimum GEF activity of Vav2 towards Rac, the localized synthesis of this phospholipid at the site of chlamydial entry was investigated, using the probe BTK-PH-GFP, where the pleckstrin homology (PH) domain of Bruton's tyrosine kinase (BTK) was fused to GFP. This domain has been demonstrated to be specific for PI 3,4,5-P_3_ [23]–[27]. Cells expressing this probe were infected by CMTPX-labeled *C. trachomatis* serovar L2, and monitored using live microscopy (Figure S2). As shown in Figure 3, localized bursts of EGFP-BTK-PH recruitment could be observed. Both the recruitment and disappearance of the fluorescent probe were rapid and transient. To test if the recruitment of BTK-PH-GFP was due to PI 3,4,5-P_3_ synthesis, transfected cells pre-treated with 100 nM wortmannin were monitored by live cell microscopy. As shown in Figure 3a and Figure S3, BTK-PH-GFP recruitment at the site of entry was not observed. Thus, BTK-PH-GFP localization at the site of entry was likely associated with PI 3,4,5-P_3_ synthesis. This localized PI 3,4,5-P_3_ synthesis would be expected to participate in the activation of Rac1 by the Vav2 GEF. That not all EBs localized with the BTK-PH-EGFP reporter could be attributed to the presence of a relatively large number of non-infectious EB particles, which is common in purified EB preparations, or that some simply do not utilize the PI3-kinase pathway of chlamydia entry.

The localized burst of PI 3,4,5-P_3_ synthesis implies the presence of the PI3-kinase at the site of chlamydia entry. Figure 3b shows that the p85 subunit of the Class I PI3-kinase interacted with the pY1 oligopeptide, but not with pY2 or the non-phosphorylated WT and F1F2 oligopeptides. In conjunction with the localized PI 3,4,5-P_3_ synthesis, the interaction of the p85 subunit implies its localization at the site of chlamydia entry.

### Vav2 and the multiprotein complex of Sos1, Eps8, and Abi1 has guanine nucleotide exchange factor activity towards Rac1

Sos1, Eps8, and Abi1 have been shown previously to act as a Rac-specific GEF, and Vav2 required binding of PI 3,4,5-P_3_ to the Dbl homology domain for optimal Rac binding and activation [19]–[22],[28]. We sought to determine if the Sos1, Eps8, and Abi1 complex and Vav2 proteins precipitated by the pY1 oligopeptide could act as a Rac GEF. Post-nuclear supernatants were prepared from HeLa cells that were depleted of Sos1 or Vav2 proteins by small interfering RNA (siRNA). As a control, lysates were prepared from HeLa cells transfected with scrambled siRNA. The levels of Sos1 and Vav2 from the respective lysates were markedly reduced. A representative Western blot of an siRNA depletion experiment is shown in Figure S4.

The different precipitates were evaluated for GEF activity towards purified His-tagged Rac1 or His-tagged Cdc42 (Table 1). His-Rac1 and His-Cdc42 molecules were mixed with the different precipitates along with ^32^P-α-GTP. An additional control in which the His-tagged Rac1 was omitted from the reaction was included. Total HeLa cell lysate showed the highest GEF activity towards the His-tagged Rac1 and Cdc42. When no biotinylated oligopeptide was present, the amount of ^32^P radioactivity was relatively low and comparable to samples in which the His-Rac has been omitted. Pulldown samples from the WT oligopeptide showed an approximately 4-fold increase relative to the samples with no oligopeptide, possibly representing the non-specific binding of proteins to the oligopeptides. However, using His-Rac1 as the target, the supernatant incubated with the pulldown samples from the pY1 oligopeptide displayed significantly elevated levels of retained ^32^P label relative to the pulldown from the WT oligopeptide for all lysates examined. An exception was the Sos1-depleted lysate, indicating the requirement for the Sos1 GEF. The pY1 precipitate from the PI 3,4,5-P_3_ -supplemented lysate showed an increase in GEF activity for both His-Rac1 and His-Cdc42 (31038 cpm vs. 51169 (Rac1) and 2821 cpm vs. 28660 cpm (Cdc42)). The corresponding increases could be attributed to the presence of Vav2, which is a GEF for Rac, Cdc42, and RhoA. Indeed, for the exchange reaction using His-Rac1 as the target, Vav2 depletion by siRNA returned the ^32^P level to that of the untreated cell lysate, even in the presence of PI 3,4,5-P_3_. While a similar reaction was not performed using Cdc42 as the target, the significantly lower level of GEF activity in the absence of PI 3,4,5-P_3_ indicate that Vav2 may be involved as well. From the data, it appears that withdrawal of PI 3,4,5-P_3_ was functionally similar to the depletion of Vav2, as this GEF appears to be inefficient in catalyzing the exchange reaction in Rac1 and Cdc42 in the absence of PI 3,4,5-P_3_. Note that the remaining GEF activity found in the Vav2-depleted lysates was likely due to the presence of Sos1, because its depletion resulted in the further 10-fold decrease in retained ^32^P label. The pY1 oligopeptide contained Sos1 and Vav2 exchange activities.

**Table 1:** Guanine nucleotide exchange activity associated with the different TARP oligopeptide coprecipitates

	None	WT	pY1	pY2	F1F2
Reg. lysate	722 (110)	3085 (455)	31038 (1840)	7634 (1155)	845 (316)
PI 3,4,5-P_3_	782 (135)	3165 (520)	51169 (3660)	31700 (2860)	857 (228)
Sos1 siRNA	846 (118)	3427 (512)	3325 (480)	3385 (720)	N/D
Vav2 siRNA+PI 3,4,5-P_3_	837 (104)	3252 (633)	34520 (2110)	870 (180)	N/D
Cdc42-His	1013 (94)	2985 (714)	2821 (813)	3842 (996)	910 (177)
Cdc42-His+PI 3,4,5-P_3_	N/D	3790 (996)	28660 (2130)	22985 (3143)	N/D
No Rac-His	962 (113)	835 (210)	844 (196)	909 (410)	924 (211)
Total lysate	161725 (12860) His-Rac1		218830 (9455) His-Cdc42		

Data are expressed as CPM Mean (S.D.).

Used His-Rac1 as the acceptor unless indicated.

N/D – not determined.

The pulldowns with pY2 yielded relatively high numbers with the PI 3,4,5-P_3_ -treated (31700 cpm) or untreated (7634 cpm) lysates. The former indicated the presence of a PI 3,4,5-P_3_ -dependent Vav2 activity, which was confirmed by the loss of retained label (870 cpm) when Vav2 was depleted even in the presence of PI 3,4,5-P_3_. Similar to pY1, the exchange reaction using Cdc42 as the target was stimulated by the addition of PI 3,4,5-P_3_, indicating the involvement of Vav2. That Vav2 depletion resulted in values that are similar to the background levels suggests that Vav2 is the only GEF towards Rac1 and Cdc42 in the pY2 pulldown. Interestingly, there was still a relatively high level of GEF activity in the pY2 pulldown from untreated lysates (7634 cpm) compared to background (722 cpm). This result was reproducible, and the significance of this observation is unclear. As expected, the oligopeptides in which the tyrosine residues have been replaced by phenylalanine failed to yield values that are statistically different from the negative controls. Taken together, the lysates pulled down by the phosphorylated peptides contained GEF activity towards His-Rac (pY1) and Cdc42 (pY1 and pY2), and that this GEF activity was due to the presence of Sos1 and Vav2, with the latter requiring the addition of PI 3,4,5-P_3_. The involvement of Cdc42 in the *in vitro* GEF reaction poses an interesting question as this GTPase has been shown not to be required in *C. trachomatis* invasion [10].

### Sos1, Eps8, Abi1, and Vav2 colocalize at the sites of chlamydial attachment

**Figure 4:**
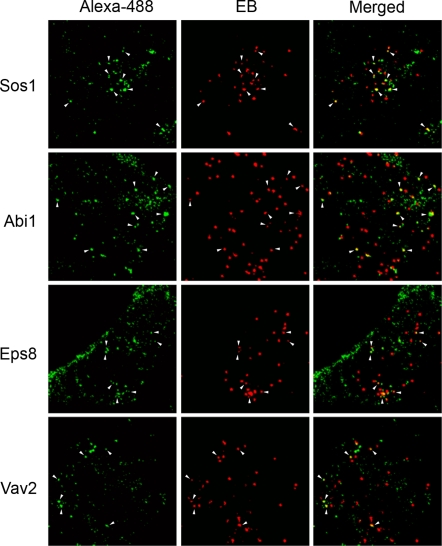
Recruitment of various host signaling molecules to the sites of entry of *C. trachomatis* L2 EBs. HeLa cells infected for 10 min were fixed and prepared for immunofluorescent staining using antibodies specific for Sos1, Abi1, Eps8, and Vav2. White arrowheads indicate protein puncta colocalizing with EBs. Bordered regions indicate areas enlarged in inset images. Scale bars = 10 µm.

Phalloidin and 4G10 antibodies also colocalize at the sites of entry, where TARP molecules are predicted to be translocated across the host plasma membrane [9],[14]. Interaction of Sos1, Eps8, Abi1, and Vav2 with TARP would result in the localization of these molecules at the sites of chlamydial entry. Indeed, colocalizations of Sos1, Eps8, Abi1, and Vav2 with invading EBs were observed by antibody staining and indirect immunofluorescence (Figure 4). All four proteins were present as distinct puncta. We observed that 30% of EBs colocalized with Sos1, 41% with Abi1, 30% with Eps8, and 24% with Vav2. The significance of these values is unclear as they could be skewed by the quality of EB preparations and the transient nature of the localization of the signaling molecules. This transient localization of the signaling molecules to the sites of entry may have prevented the visualization of some of these recruitment events in fixed cells. Another possibility is the utilization of alternate mechanisms for some of the invading EBs.

### The role of Sos1, Abi1, Eps8, and Vav2 in the invasion of non-phagocytic cells by *C. trachomatis* serovar L2

**Figure 5:**
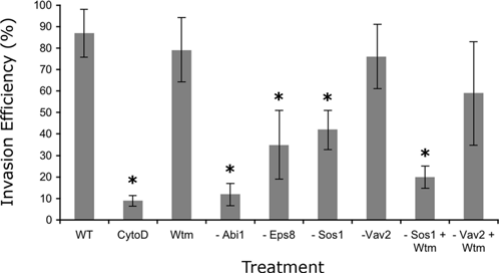
Sos1, Eps8, Abi1, and Vav2 are important for chlamydial invasion. Depletion of the four proteins under investigation resulted in significant decreases in invasion efficiency. The near wild-type levels of Vav2-depleted or wortmannin-treated cells indicate a functional compensation by the Sos1 pathway. The redundancy of the roles of the Sos1 and Vav2 pathways was demonstrated in samples depleted for Sos1 and treated with wortmannin. Results are from three independent trials with each trial done in triplicate, and represented as mean±standard deviation. Asterisks denote groups that are statisticially significantly different (p<0.05, Student's t-test) from the mock control.

Abi1 and Eps8 adaptor proteins, when in a complex with Sos1 can function as a Rac-specific guanine nucleotide exchange factor [19],[28]. Vav2 is also a well-characterized Rac GEF [20],[21]. That these proteins are found to co-precipitate with Rac and the repeated domain of TARP underscores their potential importance in chlamydial invasion. The roles of Sos1, Eps8, Abi1, and Vav2 in chlamydial entry were investigated in HeLa cells depleted individually of each protein. At 48 h post-transfection Sos1, Eps8, Abi1, and Vav2 proteins were reduced to minimal levels (Figure S4) with the knockdowns resulting in a decrease in invasion efficiency of approximately 40%, 60%, 10%, and 80% respectively (Figure 5). This suggests that Vav2 makes a very limited contribution to chlamydial invasion or that the presence of the Sos1 pathway compensates for the loss of Vav2.

To explore the possible cooperation between the two GEF activities in chlamydial invasion, Sos1-depleted cells were also treated with wortmannin, which is implicated in the Vav2-dependent activation of Rac. Figure 5 shows that the addition of wortmannin had no effect on mock-transfected cells, which would be expected if the PI_3_-kinase-independent GEF (Sos1/Eps8/Abi1) is truly functionally redundant to Vav2 with respect to chlamydial invasion. However, wortmannin treatment in conjunction with the depletion of Sos1 protein led to a significant decrease in chlamydial invasion efficiency. For comparison, cells treated with the actin filament destabilizing drug cytochalasin D were found to be the least able to support chlamydial invasion

## Discussion

Intracellular pathogens have evolved a number of different mechanisms to subvert the actin cytoskeleton and facilitate their uptake by the host cell [29]–[31]. Manipulation of the actin cytoskeleton by pathogens typically involves the modulation of the activities of host cellular proteins that participate in the complex dynamics of actin recruitment and remodeling. Some pathogens, like Salmonella and Shigella achieve this through secretion of soluble Type III effectors, while others, like Listeria directly bind host cell receptors whose signaling constitutes a cascade that regulates actin cytoskeletal remodeling [31]. Here we report the identification of the relevant GEFs that activate Rac during *C. trachomatis* invasion. Both the Sos1/Eps8/Abi1 multiprotein complex and Vav2 were found to associate with the phosphodomain of TARP in a phosphorylation-dependent manner. Optimal Rac activation by Vav2 *in vitro* also required the presence of the phospholipids PI 3,4,5-P_3_, which is generated at the site of chlamydial attachment by virtue of the interaction of the p85 subunit of PI3-kinase with the TARP phosphodomain. Localization to the sites of chlamydial entry and their functional involvement in chlamydial invasion demonstrate their importance in chlamydial invasion of non-phagocytic cells.

The multiple phosphotyrosine residues in the repeat region of *C. trachomatis* TARP are likely recognized by a number of signaling molecules containing Src homology 2 (SH2) domains, and thus act as a scaffolding to which the signaling proteins relevant to actin remodeling are recruited. The binding of the Vav2 GEF and the p85 subunit of PI3-kinase is likely due to their respective SH2 domains, which recognize phosphorylated tyrosines. There is, however, a level of specificity to this interaction as not all of the SH2 domain-containing proteins examined bound to the phosphorylated oligopeptides. For example, the adaptor protein Grb-2 failed to bind to either pY1 or pY2 oligopeptides at levels above background. Furthermore, depletion of Grb-2 by siRNA did not affect chlamydial invasion (data not shown).

**Figure 6:**
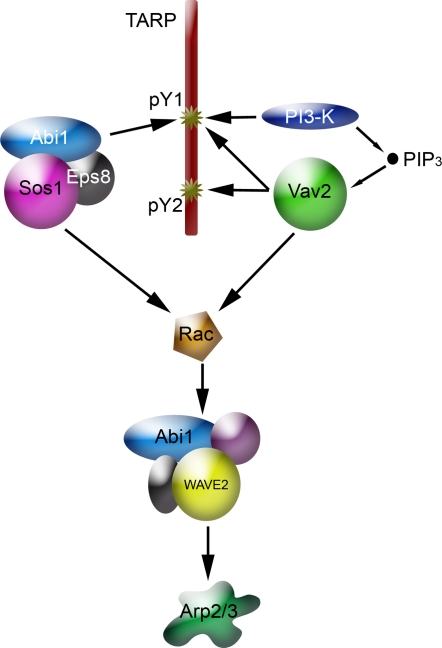
A model of the signaling pathway to the Arp2/3 complex. Rac activation, which is required for chlamydia invasion involves two different guanine nucleotide exchange factors that interact directly (Vav2) or indirectly (Sos1/Abi1/Eps8) with the phosphodomain of the TARP protein.

It has been previously shown that invasion of HeLa cells by *C. trachomatis* serovar L2 is Rac-dependent, and that WAVE2 and Abi1 are downstream effectors that activate the Arp2/3 complex [9],[10],[32]. Given this requirement for Rac activation during chlamydial invasion, the identification of the relevant Rac GEFs defines a mechanistic pathway of chlamydia invasion at the molecular level. A model of the proposed signaling pathway is shown in Figure 6.

Scita *et. al.* have previously reported the specificity of the Sos1 protein towards Rac is conferred by the Eps8 and Abi1 proteins [33]. Their report demonstrated that, while Sos1 by itself could activate both Rac and Ras, the addition of Eps8 and Abi1 shifted the specificity of Sos1 towards Rac. We confirmed that the fraction that contained Sos1/Abi1/Eps8 complex has at least a GEF activity towards Rac1. These proteins, indeed function as a complex as depletion of one of the components affected the efficiency of co-precipitation of the others. Based on this set of pulldown experiments (Figure 2c), we propose that Abi1 mediates the indirect interaction of Sos1 and Eps8 with the pY1 oligopeptide, while Sos1 and Eps8 require each other for their respective interaction with Abi1. The presence of a low level of Eps8 in pY2 in Figure 2a may be due to its interaction with Abi1, and its low level relative to that observed for pY1 was likely due to the absence of Sos1 in the pY2 fraction.

We have previously shown that expression of the dominant negative mutant of the Rac GTPase inhibited chlamydial invasion, and this inhibition correlated with the loss of actin localization at the sites of entry. The depletion of guanine nucleotide exchange factors that activate Rac, in essence is functionally equivalent to the dominant negative Rac mutant. Thus, it is likely that inhibition of the guanine nucleotide exchange factor activity towards Rac would result in the similar loss of actin localization at the sites of chlamydial invasion.

Vav2 demonstrated GEF activity towards both Rac1 and Cdc42. Our results are consistent with previous data that characterized Vav2 specificity [34]. However, the previously reported exclusive dependence of the *C. trachomatis* serovar L2 invasion on Rac1 seems to contradict our data [10],[32]. One explanation could be the lack of a sustained signal to or by Cdc42 during chlamydial invasion. Three observations appear to support this hypothesis. First, our previous data that demonstrated the lack of any detectable activation of Cdc42 could be interpreted as short-lived activation that was undetected during the time intervals chosen for the experiment [10]. Second, the synthesis of the Vav2 co-factor PI 3,4,5-P_3_ at the site of entry is extremely short-lived, resulting in the transient interaction of Vav2 with its downstream GTPase effectors. Thirdly, there is a precedent for a difference in the duration of signals transduced by GTPase protein. An excellent example is the difference in Rac and Ras signaling, in which Ras signaling, mediated by the Sos1/Grb-2 complex during growth factor stimulation, was short-lived relative to signaling by Rac, which was mediated by Sos1/Abi1/Eps8, under similar conditions of growth factor stimulation [35]. Thus, it is quite possible that the transient production of PI 3,4,5-P_3_ may explain the apparent lack of Cdc42 involvement in chlamydial invasion. On the other hand, Rac1 could be activated by two distinct pathways; one of which is independent of PI 3,4,5-P_3_. This Rac activation pathway would be expected to predominate during conditions of high PI 3,4,5-P_3_ turnover. The presence of a PI 3,4,5-P_3_ -independent pathway of Rac1 activation would be expected to confer a wortmannin-insensitive invasion mechanism. Indeed, entry of serovar L2 is insensitive to treatment with 100 nM wortmannin. During inhibition of PI 3,4,5-P_3_ synthesis, the Sos1/Abi1/Eps8 pathway of Rac activation remains functional, with the host cell still supporting chlamydial uptake.

We observed highly localized and transient bursts of synthesis of PI 3,4,5-P_3_ at the sites of chlamydial entry. This implies the recruitment of the regulatory subunit (p85) of PI_3_-kinase and its binding partner, the catalytic (p110) subunit. Indeed, the p85 subunit was found to bind preferentially the pY1 oligopeptide. In conjunction with the observed interaction of Vav2 with the pY2 oligopeptide, these two components may bind independently to two distinct binding sites, but cooperate to activate the Vav2-dependent pathway. That the PI3-kinase p85 subunit displayed the same preferential binding to pY1, as the Sos1/Abi1/Eps8 complex is suggestive of a competition or a hierarchical control that possibly determines which pathway is utilized for chlamydia uptake. The reproducible decrease in invasion efficiency during depletion of the Sos1 protein indicate the preferential utilization of this pathway compared to the Vav2 pathway. In addition, because TARP homologs within the *C. trachomatis* species have at least two phosphodomains, it is intriguing to speculate that the domains cooperate with each other to recruit signaling molecules, and bring into proximity components to stabilize the signaling complex and/or transduce the desired signal. However, the presence of multiple potential binding sites adds another level of complexity to this signaling process in the regulation of this signaling pathway.

Chlamydophila species that possess TARP homologs, but do not contain the repeated phosphodomain clearly demonstrate that alternative mechanisms of actin remodeling and recruitment exist. An attractive scenario is the requirement for an additional bacterial factor that interacts with a conserved region of TARP and substitute for the tyrosine phosphorylation during recruitment of signaling molecules. The enterohemorrhagic E. coli (EHEC) EspFu/TccP protein directly binds to the GTPase binding domain of N-WASP to induce pedestal formation independent of Tir tyrosine phosphorylation and Nck recruitment [36]. Whether a similar mechanism is at work in other species of chlamydia is certainly a topic that warrants further investigations.

Equally important is how the actin nucleating function of the conserved C-terminal domain of TARP fit with the model that requires signaling to the Arp2/3 complex to activate its actin-nucleating activity. It is unlikely that the Arp2/3-independent actin nucleating activity of the C-terminal domain of TARP is sufficient for chlamydia-induced actin recruitment and invasion. It has been reported that the invasion of *C. trachomatis* serovar L2 depends on Arp2/3. A model that we favor is a cooperative one, in which the activation of the Arp2/3 complex by the Rac-dependent signaling pathway initiates actin nucleation forming short actin filaments. These nascent filaments are then bound by the WH2 motifs within the C-terminal domain of TARP, nucleating spontaneous actin polymerization. This model fits a number of critical observations – a) *C. trachomatis* invasion is Arp2/3-dependent; b) actin polymerization can be mediated by a minimal TARP domain that contains the WH2 motifs in an Arp2/3-independent manner; and c) the C-terminal domain has a higher affinity for F-actin. How these two mechanisms cooperate is under investigation.

In summary, this report is the first to directly implicate the *Chlamydia trachomatis* Type III effector TARP in invasion by identifying the host signaling molecules that link TARP to the actin remodeling machinery. The potential for TARP to be differentially phosphorylated at the two tyrosine residues described in this report and the presence of multiple phosphodomains together imply the presence of a control mechanism that fine tunes the function of TARP in chlamydia invasion. This modulation may be at the level of phosphorylation, binding of the signaling complexes, their respective stability, or the ability of the signaling pathway to cooperate with the nucleating function of the C-terminal domain of TARP. Clearly, many questions still need to be answered to gain a full understanding of the invasion process of this medically important obligate intracellular pathogen.

## Materials and Methods

### Organisms and cell culture

*C. trachomatis* serovars L2 (LGV-434) were grown in and harvested from HeLa 229 cells as previously described [37]. EBs used for infections were purified by Renografin (E. R. Squibb and Sons, Princeton, NJ) density gradient centrifugation. Fluorescent CMTPX-labeled EBs were prepared as described previously [38], with slight modifications [10].

### Antibodies, constructs, and siRNA

Antiphosphotyrosine monoclonal antibody (MAb) clone 4G10 and FITC-conjugated 4G10 were purchased from Upstate USA (Waltham, MA); anti-Sos1 monoclonal, anti-Abi1 monoclonal, anti-p85, and anti-Eps8 polyclonal antibodies were from and Abcam; anti-Vav2 rabbit polyclonal antibody was from Zymed (South San Francisco, CA); and anti-Rac1 monoclonal antibody was purchased from Cytoskeleton. Secondary antibodies for immunoblotting were horseradish peroxidase-conjugated anti-mouse or anti-rabbit (Cell Signaling Technology, Inc., Beverly, MA). GFP-actin (from Dr. S. Grieshaber, University of Florida), GFP-BTK-PH (from Dr. J. Celli, LICP, NIAID) and GFP-Rac (from Dr. M. Way, Cancer Research UK, London, UK) were described previously [39],[40]. siRNAs against human Sos1#16747, #16656, and #16561), Eps8 (#107412, #107411, and #14635), Abi1 (#137945, #137944, #137946), and Vav2 (#13196, #214755, #138982) were purchased from Ambion.

### Cloning

Synthesis of pEGFP-C1 L2 TARP was performed as follows. The full length L2 TARP gene was amplified using KpnI linker-containing FWD primer 5′-ATGGTACCATGACGAATTCTATATCAGGTG-3′ and the BamH1 linker-containing reverse primer 5′-ATGGATCCTGTTCTCCTTTGCTTTTTA-3′ with the PCR product digested with KpnI (New England Biolabs) and BamH1 (New England Biolabs) for an overnight ligation into the pEGFP-C1 vector (Clontech), which was pre-digested with the same restriction enzymes and dephosphorylated using calf intestinal alkaline phosphatase (New England Biolabs). Ligation was performed at 15°C for 16 h.

The N-terminal domain of CD4 (amino acids 1–372) was subcloned from pCMV-Sport XL5-CD4 purchased from Origene by PCR using the primers 5′-CACCATGAACCGGGGAGTCCCTTTT-3′ and 5′-AAGCTTCTTCTACGGATCGGGTTACTT-3′ into the pcDNA3.1 TOPO vector (Stratagene, Carlsbad, CA). The resulting vector was digested with Kpn1 to accommodate the PCR fragment containing one repeated unit (amino acids 174–222) of the TARP phosphodomain region. This PCR fragment was generated by the amplification of the pEGFP-C1 L2 TARP using the primers 5′-ATGGTACCCTTCAGAAAGCTCAGAAACTA-3′ and 5′-ATGGTACCGTAGGAGGAGCCTCTTAGA-3′ containing Kpn1 linker sequences. The orientation of the insert relative to the the CD4 reading frame was determine by colony PCR using the primers CD4 forward primer 5′-CACCATGAACCGGGGAGTCCCTTTT-3′ and 5′-CTTAGTCATCAATACTCTCATAAATATTTTCAGTAGTGCTTATCG-3′, which annealed to an internal region of the 1xR sequence.

### Transfection

HeLa 229 or Cos7 cells were seeded on 12-mm glass coverslips in 24-well plates to obtain a monolayer of approximately 50% confluence. Transfections of plasmid constructs were performed using FuGene (Roche, Indianapolis, IN) according to the manufacturer's instructions. The transfection mixture was prepared as follows. The FuGene reagent (3 µl was diluted into 97 µl Optimem (Invitrogen) serum-free media, and to which DNA (1.0 µg) and added. After a 20-minute incubation at room temperature, the complexes were added to 1 well of a 24-well plate containing 100 µl of Optimem. The transfection cocktail was incubated at 37°C. After 4 hours, the transfection medium was removed and antibiotic-free RPMI media with 10% fetal bovine serum was added. Expression from the transfection vectors was allowed to proceed for 24 hours at 37°C.

siRNA transfection with the transfection reagent Ribojuice was performed per the manufacturer's instructions. Briefly, 5 nM siRNA was incubated with 4 ul (for a well in a 24-well plate) or 100 ul (for a 100 mm dish) of Ribojuice in OPTI-MEM and incubated at room temperature for 30 min. HeLa cells at 80% confluency were washed once with 1× HBSS and incubated in 100 ul or 1 ml OPTI-MEM. The siRNA transfection solution was added to the cells and incubated for 4 h. The transfection medium was removed and replaced with complete RPMI (10% FBS, L-glutamine, and gentamicin). At 48 h post-transfection, the levels of the proteins of interest were evaluated by Western blot.

### Oligopeptide pulldown

Briefly, post-nuclear supernatants were prepared as described previously [32], and divided into five aliquots of 0.75 ml each. Each aliquot was incubated with 10 µM of one type of oligonucleotide (Sigma-Genosys) for 1 h at 4°C with rocking, followed by an additional hour of incubation with 20 µl of streptavidin-coated paramagnetic beads (Dynal). The beads were removed from suspension with a magnet (Dynal), and washed three times with cold RIPA buffer. The precipitated fractions were suspended in 150 µl of Laemmli buffer and boiled prior to gel electrophoresis.

### SDS-PAGE and immunoblotting

Proteins were separated on a 10.5–14% continuous gradient sodium dodecyl sulfate-polyacrylamide gel electrophoresis (SDS-PAGE) gels (Bio-Rad, Hercules, CA) and transferred to a 0.45-µm Trans-blot nitrocellulose membrane (Bio-Rad). Immunoblots were developed using Super Signal West Femto chemiluminescence reagent (Pierce Biotechnology, Rockford, IL) per the manufacturer's instructions.

### GDP-GTP exchange assay

The oligopeptide pulldown fractions described above were subjected to an *in vitro* GDP-GTP exchange assay [41]. 25 µCi of [α-^32^P]GTP (3000 Ci/mmol, Amersham) and 83 pmol of cold GDP (10-fold excess over labeled), and 1 µg of Rac-His or Cdc42-His (Cytoskeleton) were added to 200 µl of exchange buffer (50 mM HEPES, pH 7.5, 1 mM MgCl_2_, 1 mM Dithiothreitol, 100 mM KCl, and 0.1 mg/ml bovine serum albumin). 10 µl of this solution was added to the oligopeptide pulldown fraction. The reaction was incubated at room temperature for 30 min, and terminated by the addition of 1 ml of the stop buffer (50 mM HEPES, pH 7.5, 5 mM MgCl_2_, 1 mM DTT, 10 ug/ml BSA, and 0.1 mM GTP) and immediate incubation on ice. The beads containing the oligopeptide and associated proteins were pelleted (30 sec at 15,000 rpm), and the supernatant were loaded onto nitrocellulose filters using a vacuum manifold (Bio-Rad). The filters were washed three times with ice cold PBS with 5 mM MgCl_2_. Radioactivity retained on the filters was counted by scintillation.

### Immunofluorescence microscopy

Cells grown on coverslips were fixed in freshly prepared 4% paraformaldehyde in PBS for at least 1 h at 4°C. If required, the fixed cells were permeabilized with 0.1% Triton X-100 in PBS for 2 min at RT. The permeabilization buffer was removed and the cells rinsed three times with 1× PBS. Primary antibodies were diluted to their respective working concentrations (α-Sos1 1∶250 (Abcam), α-Abi1 1∶1000 (Abcam), α-Eps8 1∶250 (Abcam), α-Vav2 1∶500 (Zymed), α-L2 EB, 1∶1000 from Ted Hackstadt, RML, NIAID), added to the fixed cells, and incubated at 4°C overnight. Anti-rabbit or anti-mouse IgG secondary antibodies used were conjugated to either Alexa 488 or Alexa 594 (Invitrogen). Coverslips were mounted using Mowiol on glass slides, and samples visualized using the Olympus Fluoview 500 Laser Scanning Microscope. Images were processed using Adobe Photoshop Creative Suite.

### Live cell microscopy

Cells grown on Delta T culture dish (0.17 mm thick) (Bioptechs) overnight and transfected with a GFP-BTK-PH expression construct. At 18 h post-transfection, the cells were infected with CMTPX-labeled *C. trachomatis* LGV serovar L2 EBs, and observed at 5 s interval using an UltraView Live Cell Imaging system fitted with a Bioptechs Delta T4 objective heater. Images were assembled into a time lapse Quicktime movie using NIH ImageJ (Rasband, W.S., ImageJ, U. S. National Institutes of Health, Bethesda, Maryland, USA, http://rsb.info.nih.gov/ij/, 1997–2007.)

### Invasion assay

Assay for invasion was performed as described previously [7]. The experiments were performed three independent times, in triplicate each time. The Student's t-test was used to determine statistical significance.

## Supporting Information

Figure S1 Scanning electron micrograph of cells mock-treated (left), treated with 4 µM cytochalasin D (middle), and 30 min after cytochalasin D has been removed. Removal of the F-actin-destabilizing drug resulted in the preferential reformation of microvilli at the sites of chlamydia attachment, indicating the induction of signaling directly underneath the EBs and the restriction of lateral mobility of the signaling complex. Scale bar = 1 µm.
(2.04 MB TIF)

Figure S2 Localized synthesis of PI 3,4,5-P_3_ at the sites of chlamydial entry. Cos-7 cells expressing the pleckstrin homology domain of Bruton's tyrosine kinase fused to green fluorescent protein (BTK-PH-GFP) were monitored by live cell microscopy upon infection with CMTPX-labeled *C. trachomatis* serovar L2 elementary bodies. Images were acquired at 5 s intervals and compiled using the NIH Image J software. Note that the recruitment of the fluorescent probe is short-lived, indicating the transient nature of the signaling cascade. It is important to note that not all of the EB particles induced the synthesis of PI 3,4,5-P_3_ because there likely were non-infectious particles in the EB preparations. Loss of infectivity occurs during harvest, purification, and labeling of elementary bodies. A) Mock-treated cells; B) Wortmannin (100 nM)-treated cells.
(3.72 MB MOV)

Figure S3 Wortmannin (100 nM) treatment of Cos-7 cells expressing the BTK-PH-GFP probe abolished recruitment of the probe at the sites of attachment of CMTPX-labeled *C. trachomatis* serovar L2 elementary bodies. Images were acquired at 5 s intervals and compiled using the NIH Image J software.
(3.57 MB MOV)

Figure S4 Representative Western blots demonstrating the efficiency of depletion by siRNA of proteins of interest. HeLa cells were mock-transfected, transfected with a scrambled siRNA or cocktail of three siRNA for each target. Protein levels were analyzed by Western blot at 48 h post transfection.
(1.01 MB TIF)
