# Supplementary figures and images for: Chlamydial Entry Involves TARP Binding of Guanine Nucleotide Exchange Factors
Source: PLoS Pathog. 2008 Mar 7;4(3):e1000014. doi: 10.1371/journal.ppat.1000014 (PMC2279300; doi:10.1371/journal.ppat.1000014)

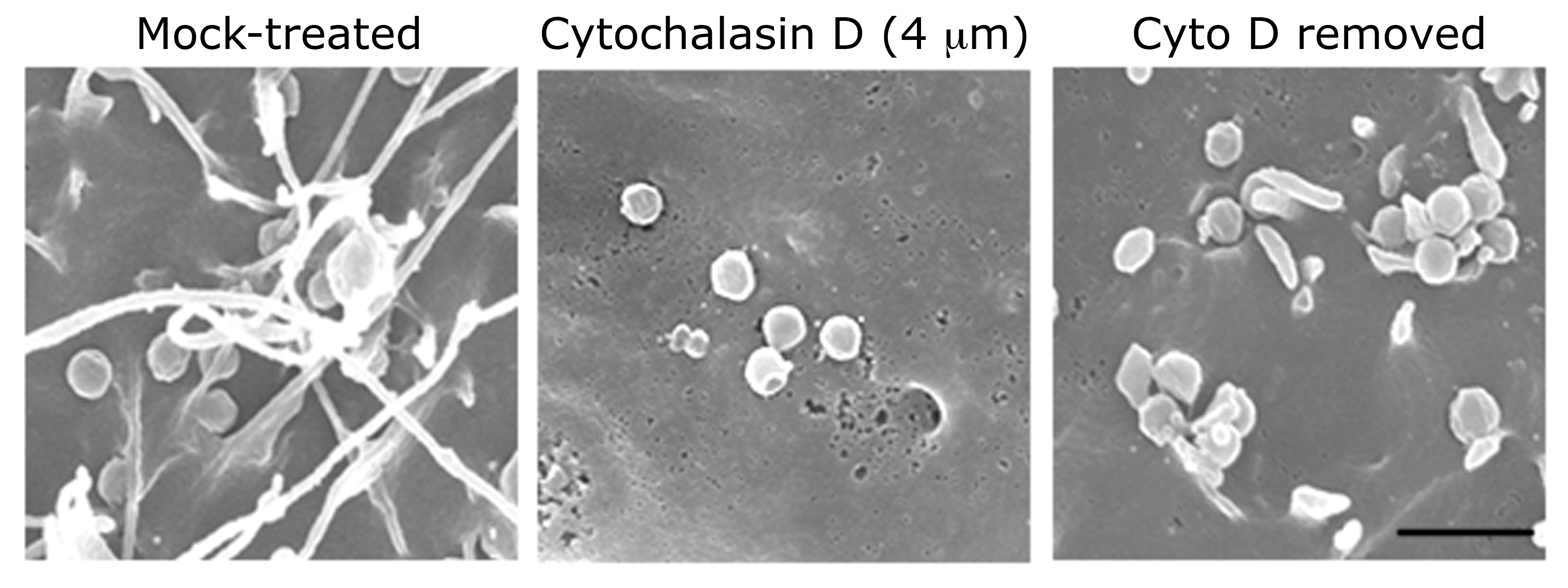

Supplement: Figure S1 — Scanning electron micrograph of cells mock-treated (left), treated with 4 µM cytochalasin D (middle), and 30 min after cytochalasin D has been removed. Removal of the F-actin-destabilizing drug resulted in the preferential reformation of microvilli at the sites of chlamydia attachment, indicating the induction of signaling directly underneath the EBs and the restriction of lateral mobility of the signaling complex. Scale bar = 1 µm. (2.04 MB TIF) [file ppat.1000014.s001.tif]

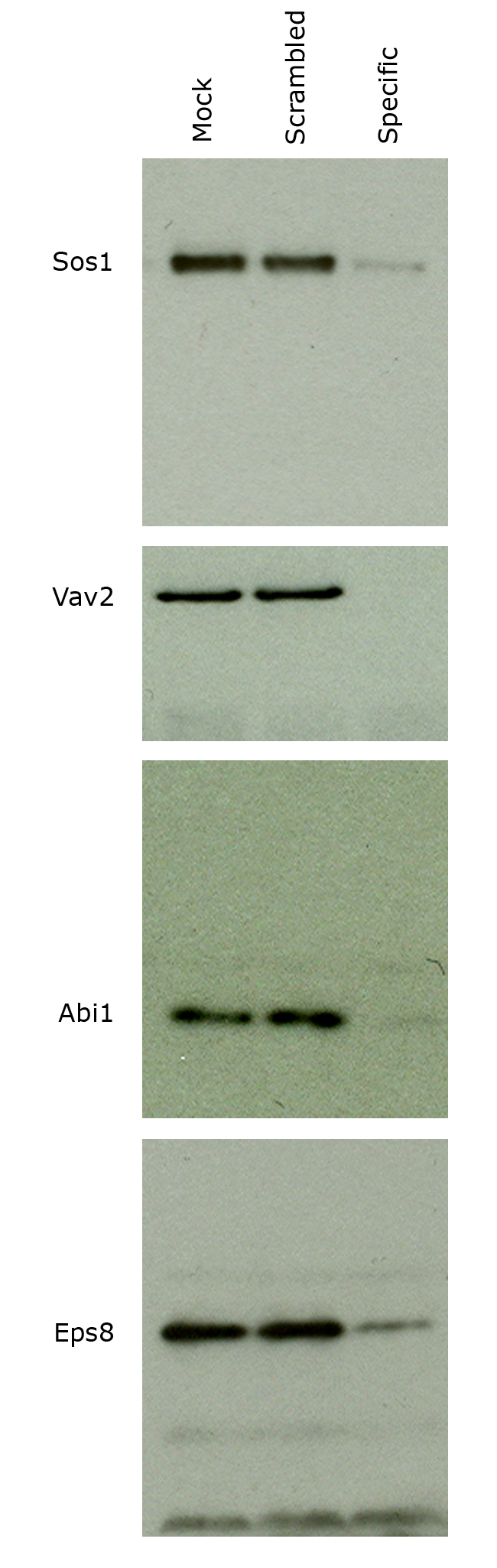

Supplement: Figure S4 — Representative Western blots demonstrating the efficiency of depletion by siRNA of proteins of interest. HeLa cells were mock-transfected, transfected with a scrambled siRNA or cocktail of three siRNA for each target. Protein levels were analyzed by Western blot at 48 h post transfection. (1.01 MB TIF) [file ppat.1000014.s004.tif]
